# Spray Drying for the Encapsulation of Oils—A Review

**DOI:** 10.3390/molecules25173873

**Published:** 2020-08-26

**Authors:** Nameer Khairullah Mohammed, Chin Ping Tan, Yazid Abd Manap, Belal J. Muhialdin, Anis Shobirin Meor Hussin

**Affiliations:** 1 Department of Food Science, Faculty of Agriculture, Tikrit University, Tikrit 34001, Iraq; nameer@tu.edu.iq; 2 Faculty of Food Science and Technology, Universiti Putra Malaysia, Serdang, Selangor 43400, Malaysia; tancp@upm.edu.my (C.P.T.); myazid@upm.edu.my (Y.A.M.); belal@upm.edu.my (B.J.M.); 3 Halal Products Research Institute, Universiti Putra Malaysia, Serdang, Selangor 43400, Malaysia

**Keywords:** spray drying, encapsulation, oils, wall material, food ingredients

## Abstract

The application of the spray drying technique in the food industry for the production of a broad range of ingredients has become highly desirable compared to other drying techniques. Recently, the spray drying technique has been applied extensively for the production of functional foods, pharmaceuticals and nutraceuticals. Encapsulation using spray drying is highly preferred due to economic advantages compared to other encapsulation methods. Encapsulation of oils using the spray drying technique is carried out in order to enhance the handling properties of the products and to improve oxidation stability by protecting the bioactive compounds. Encapsulation of oils involves several parameters—including inlet and outlet temperatures, total solids, and the type of wall materials—that significantly affect the quality of final product. Therefore, this review highlights the application and optimization of the spray drying process for the encapsulation of oils used as food ingredients.

## 1. Introduction

Modern consumers are concerned about their health, and this concern leads to a high demand for foods that contain bioactive or functional ingredients (especially natural ones) that increase the nutritional value and health status of food [1]. In particular, there is a growing demand for nutritious and healthy oils in the food, pharmaceutical, and cosmetic industries due to their multi-functional properties. Nevertheless, due to their high level of unsaturation, vegetable and marine oils are prone to oxidation deterioration in addition to the resulting production of unpleasant taste. Moreover, oils are unstable under processing and storage conditions due to their sensitivity to light and heat, which limits their application in the food industry. Hence, it is necessary to protect the oils to improve their stability during handling, processing, and storage [2,3]. These fluctuations negatively affect the developed products in terms of their shelf-life, sensory properties, and overall acceptability [4]. Encapsulation is a promising approach that is widely used to overcome the above-mentioned problems by protecting the core materials from heat, light and oxygen, thus promoting stability, increasing bioavailability, flavour-masking, and controlled release, while maintaining the oils’ functional properties and increasing their ease of handling [5]. Encapsulation can be defined as a process for entrapping one substance (termed the core material or active agent) within another substance (the coating, shell, or carrier/wall material). Encapsulation using spray drying is a reliable technique in the food industry that has been successfully used to overcome these challenges [6,7]. Microencapsulation technology is becoming a common technology used with oils around the world, such as microencapsulated palm oil, microencapsulated fish oil, and microencapsulated coconut oil, which are used as food ingredients.

Encapsulation may enhance ease of handling, adequacy of concentration and uniformity of dispersion [8]. Despite the rapid drying contact time (several seconds) of the core materials in the drying chamber, spray dryers have high drying temperatures, typically with an input air temperature of 150 to 250 °C, and an outlet air temperature of 50–80 °C [9]. The resulting particles range from a few nanometers to a few hundred micrometers [10]. The structural type and desired size generally depend on the preparation method and the wall materials applied [11]. The application of encapsulation in food production requires the wall materials to be of food grade and have protective properties for the core materials against external factors. The carrier materials typically used for oil encapsulation include synthetic polymers and natural biomaterials (commonly carbohydrates and proteins) [12]. Therefore, this review focus on the principles, wall materials, and processing parameters of spray drying for oils encapsulation. In addition, this review illustrates recent research on oil encapsulation through spray drying and the application of these products in foods.

## 2. Oil Encapsulation Benefits

Encapsulation of oils improves the oxidative stability of their lipids and protects sensitive constituents (core) such as active compounds, oils, flavour compounds, and vitamins from environmental factors. This facilitates high solubility and easy mixing of the core material, and manages the release of the core material in order to attain an appropriate delay until the appropriate stimulus. Moreover, it reduces the evaporation of volatile compounds in the core material, masking or covering any unpleasant tastes associated with the core material [13,14,15,16]. The reasons for applying encapsulation for oils can be summarized as follows:

(a) Improvement of the oxidative stability of lipids by applying the encapsulation process to produce powdered edible oil products, helping to prolong shelf-life by protecting oils from oxidation [14].

(b) Shielding of core materials, which are usually sensitive compounds such as oils, flavours, and vitamins, from oxygen, light, or water. In general, food oils exhibit significant susceptibility to light, temperature, air, and irradiation [17,18].

(c) Alteration of the oils from a liquid to a dry form in order to produce powder with high solubility and acceptable mixing properties of the core materials. Conversion of the fluid feed (flavours and edible oils) into powders in solid form with desirable handling properties is an important use of encapsulation in the food industry [19].

(d) Managing the release of the core material in order to achieve a suitable delay for the proper stimulus; a vital benefit of the encapsulation of oils and flavours is control over the release time of the active ingredients until they have arrived at their target [20].

(e) Preventing evaporation of volatile compounds in the core materials. The dry powder obtained through the encapsulation process possesses high oxidation stability and reduced volatility. The encapsulation of oils results in a dry powder with improved oxidation and less volatility, which simplifies its application in various end-products such as cakes and beverages [21].

(f) Masking or covering the unpleasant tastes of the core material. The incorporation of some edible oils, such as vegetable oils or marine oils, in food products is in high demand due to the nutritional value of these products [16]. In addition, encapsulation can also help to overcome the main problems associated with food containing 3 PUFA, the unpalatable “fishy” flavour of fish oil, and the susceptibility of polyunsaturated fatty acids to oxidation, has all of which have a negative impact on food acceptability [16,22]. Encapsulation positively affects the stability and resistance of oils under storage conditions in comparison to oils without encapsulation [23].

## 3. Encapsulation Using the Spray Drying Technique

**Figure 1:**
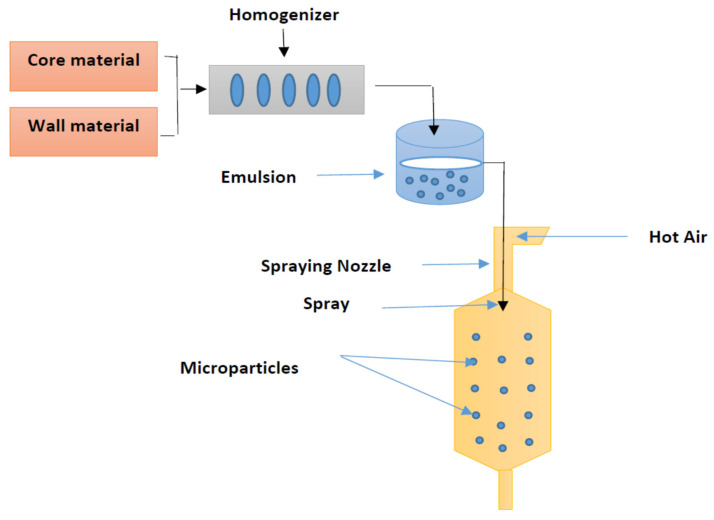
Schematic representation of the encapsulation process by spray-drying.

Encapsulation is a technique whereby small particles or droplets of active substances that have several attractive characteristics are enclosed within a coating in order to protect them against environmental factors such as oxygen, light, moisture and interactions with other compounds. Encapsulation is typically used to obtain particles with a diameter of 1–1000 μm of food ingredients or other materials. In addition, this technique can enable the controlled release of the encapsulated core when certain conditions are met [24]. Encapsulation using a spray dryer has frequently been used for food production on an industrial scale since the late 1950s, primarily for fats and oils, and flavourings and colourings [25]; see Table 1. Moreover, encapsulation is widely used in the food industry to incorporate oil aromas in a spray-dried form, because it is inexpensive, flexible, can be used in continuous operation, and produces particles of good quality [5]. The process can be conducted by changing the slurry emulsion from a liquid form into a powder in a continuously operating procedure. The basic principle of this method is to dissolve the core/wall materials in water to prepare an emulsion in liquid form, and then to feed this emulsion into a hot medium (100–300 °C) to evaporate the water. The final dried product can be collected in powder form, or as agglomerated particles, depending on the nature of the materials used in the feed, the design of the dryer’s operation, and the operating conditions. The high temperature of the drying chamber facilitates the water evaporation from the droplets [26]. Despite the energy used in the spray-drying process in terms of heat, spray drying has been shown to be more efficient than freeze drying for the encapsulation of oils, with a 30–50 times lower cost [27]. This technique does, however, require high-temperature conditions and access to air. Although the temperature of the spray dryer is high, the wet-bulb only requires a short duration of exposure (few seconds), and water vaporization will take place in the range of 30 to 50 °C [28]. A schematic diagram of the encapsulation process using the spray drying technique is presented in Figure 1. According to Bakry et al. [18], encapsulation using spray-drying consists of four main stages: (i) preparation of a stable emulsion; (ii) homogenization of the dispersion; (iii) atomization of the emulsion; and (iv) dehydration of the atomized particles. Typically, the first stage is carried out by dissolving the wall materials in distilled water and emulsifying, or dispersing using a magnetic stirrer overnight at 25 °C to ensure full saturation of the polymer molecules, and to prevent any variations caused by temperature changes. Before starting the second stage, core materials mixed with an aqueous solution of the wall materials and then the emulsifying agent can be added, depending on the emulsifying characteristics of the wall materials. The formed emulsion containing the wall materials and the core substances must be stable until the drying stage [29].

## 4. Unit Operation of Spray Drying

The process of spray drying is illustrated in Figure 1. The steps of the spray drying process involve: (a) atomization of fluid feed; (b) drying of the medium; (c) drying of the feed and spray contact; and (d) separation of the product from air. The properties of the final product are directly influenced by all of the steps, including their operational parameters. Spray drying is a very rapid and reproducible drying method, due to the very large surface area created by the atomization of the liquid feed [30].

### 4.1. Feed Atomization

Atomization involves converting the fluid feed (emulsion) into small droplets of uniform size resulting in a balance of heat and mass transfer in the drying stage, as well as increasing the surface area and allowing a good distribution of the feed within the dryer chamber. The size enlargement of the particles (surface area) leads to fast water evaporation and formation of a crust, drying the feed in seconds. Different types of atomizers, including rotary atomizers, pressure nozzles, pneumatic nozzles and sonic nozzles, exist to accomplish feed liquid collapse [30]. To choose the atomizer type based on the feed needing to be converted to dried powder and also on the desired particle size of the final product.

### 4.2. Air Flow Contact

Air flow contact is influenced by the fluid spray and the air contact time, because both factors determine the drying rate and the intensity of the drying. At this stage, the air flow contact starts from the drying step and continues during atomization. The atmosphere inside the heating chamber of the spray dryer is controlled by the operating parameters of the air heating and filtration system. The gas used during the process is usually nitrogen or another gas chosen based on the sensitivity or instability of the feed in the presence of oxygen [30]. In general, two types of feed-drying air flow conditions are available:

(a) The co-current drying design: in this configuration, the atomizer is located in the top of the drying chamber along with the drying gas stream inlet. The feed is sprayed in the same direction as the hot air, and thus it is preferred for heat sensitive composites. The inlet temperature is usually 150–220 °C and rapid vaporization takes place; the outlet temperature is 50–80 °C, which limits the thermal degradation. This design exposes heat-sensitive materials to the lower exit air temperature only. 

(b) The counter-current drying design: this system is not popular and has limited applications for dry products. The emulsion and the drying air are introduced at opposite ends of the dryer. Although this configuration is more thermally efficient than the co-current drying design as it exposes the dried powders to high temperatures, it has limited use for products that are sensitive to heat. As a result, of the complicated flow, the drying phenomena are not clear in these systems [29,31].

### 4.3. Drying and Particle Formation

The third step is drying and particle formation by convection. The heat is transferred from the drying medium to the droplets, where it is converted to latent heat during evaporation of moisture content from the droplets. The temperature and mass transfer rate are based on the diameter of the droplets and the relative velocity of the air and the droplets. Quick moisture evaporation immediately happens when the droplets come into contact with the drying air. Initially, the droplet surface exhibits a continuous mass transfer of moisture from within the droplet. At last, a dried shell is formed and moisture evaporation continues at a slower rate until the final product is formed [29,30].

### 4.4. Separation of Product from the Drying Air

The last step involves the separation of the product from the drying air. The spray drying begins when the dried particles fall to the bottom of the drying chamber and are collected through gravitational effects; while fines entrained in the drying air are parted from the atmosphere and separation occurs after the dried particles recovered in a cyclone filter that is located outside the dryer with collection bottle [29]. The liquid emulsion consists of the wall and core materials and is converted through the spray drying process into dried particles in powder form. The dried particles are spherical in shape with a size range from 10 to 100 micrometres [32].

## 5. Optimizing the Encapsulation Process Conditions

To obtain high encapsulation efficiency and the desired particle quality, the spray drying parameters should be optimized, even if the coating material is appropriate. The encapsulation efficiency is directly influenced by the characteristics of the wall/core materials, the properties of the in feed emulsion and the parameters of the spray drying process, including inlet/outlet air temperature, humidity, air flow rate and the type of atomization [33].

### 5.1. Inlet and Outlet Temperatures

To obtain a final product with a high yield and degree of encapsulation, the inlet and outlet air temperatures should be optimized, and the feed emulsion should be stable throughout the processing time [34]. In general, the inlet air temperature is in the range of 150–220 °C and evaporation occurs instantaneously. The low air inlet temperature results in a low evaporation rate, which leads to microcapsules with high-density membranes, high moisture content, low fluidity, and ease of agglomeration. Therefore, the particles will easily stick to the internal wall of the drying chamber, resulting in a low yield. However, too high an inlet temperature results in extreme vaporization, and membrane cracks may occur, and subsequently premature release and degradation or loss of encapsulated cores [29]. According to Carmona [35], optimizing the encapsulation of palm fibre oil by spray drying and the range of inlet air temperature was (130–202 °C). The optimum inlet temperature was found to be 166 °C. Recently, Başyiğit, B., et al. studied the effect of inlet air temperature (120–220 °C) on the properties of sour cherry oil encapsulated using spray drying [36]. The researcher observed that higher inlet air temperature positively affected the flowability and the optimal temperature was 195 °C. The author suggested that the effects were due to the wide range of inlet and outlet temperatures reported, which is considered to be a crucial parameter in the spray drying process to have particles with stable characteristics.

### 5.2. Total Solids of the Emulsion

The total solids concentration refers to the (wall material + oil) ratios in the emulsion calculated and represented on a dry basis. Jafari et al. [15] reviewed a variety of previous studies that optimized total solids in the emulsion for encapsulation. The review recommended maximum feed solid content to be applied. However, other studies suggested that the optimum feed solid content should be used for food flavours and oils due to two facts: first, using high content of wall material overrides solubility. Hence, un-dissolved wall materials will not improve the encapsulation process and will result in lower flavour retention for the dried particles. The second reason is to achieve the optimum emulsion, which is correlated with the viscosity of the primary emulsion. The character of the core material affected the emulsion content of the total solids. The total solid content has a pronounced influence on the core materials that are most susceptible to loss, like volatile compounds, as well as the efficiency of encapsulation. Recently, Frascareli et al. [37] studied the encapsulation process conditions of coffee oil encapsulated by spray drying, and the optimized total solids was 30%. In another study, by Carmona [35], the effect of total solid content (20–40%) on characteristics of spray-dried palm fibre oil was investigated, and the optimum solid content was 35%.

In addition, Ng et al. [38] demonstrated that 40% total solid content (wall/oil) was the most effective formulation for the encapsulation efficiency (MEE) and the oxidative stability of encapsulated kenaf seed oil. Moreover, the optimum total solid reported by [36] was found to be 20% for the sour cherry oil encapsulated by spray drying when optimization was conducted with the range of total solid (16.59–33.41%). Therefore, total solid concentration is a very important factor that needs to be covered when obtaining encapsulated oils with desired properties. Table 1 summarizes the optimization of encapsulation conditions of several oil sources. The optimized spray-drying conditions for various ingredients within different wall materials were shown to be different. The total solids were in the range 20–40%. Moreover, the range of the inlet air temperatures was 135–202 °C.

### 5.3. Wall Materials

The purpose of the wall materials is to act as a barrier between the core materials and any external factors that may cause their deterioration, to inhibit premature interactions between the core material and other ingredients, to reduce the reactivity of the core material with regard to the external environment, to limit volatile losses, and also to enable controlled or sustained release under desired conditions [29].

Table 2 shows different types of wall materials used in spray drying of oils [58]. The selections of the encapsulation technique and wall materials are interdependent. Wall material is very important for the oil encapsulation stability and protection efficiency of the core compound. The wall material influences the emulsion stability and the characteristics of the resulting microcapsules [59,60]. An ideal wall material should be highly water soluble and of low viscosity, while also possessing film forming properties. In addition, wall materials should have sufficient emulsifying ability to produce stable emulsions prior to spray drying [29]. However, a wide variety of encapsulating materials have been used for the encapsulation of flavours and oils, including low molecular weight polysaccharides (starches, maltodextrins (MD), gum Arabic (GA) and corn syrups), lipids (mono and diglycerides) and proteins (casein, milk serum and gelatin), as well as new emerging biopolymers, such as Millard reaction products [61] . Mixing two or more agents together may result in favourable characteristics such as maximizing the encapsulation efficiency of spray-dried powders and having an excellent stability and droplet size distribution for the emulsions [39].

#### 5.3.1. Carbohydrates

Starches that have been oxidized or incorporated with lipophilic groups have generally been found to have good solubility and good emulsifying and oil retention properties with low viscosities at high solid concentrations. However, when these materials are used individually, they lack the interfacial properties needed for high encapsulation efficiency, and are thus blended with other encapsulating materials such as proteins or gums [60]. Improving the encapsulation properties of wall materials can be achieved by chemically modifying carbohydrates. For instance, some modified starches have surface active properties and are widely used in the process of encapsulation by spray-drying. Hydrolysed starch products are hydrophilic compounds, and thus have little affinity for hydrophobic flavours and oils [62,63].

Maltodextrins are starch hydrolysates that are produced via partial hydrolysis of starch using either acidic or enzymatic processes. Maltodextrin is popular in food processing, because it is inexpensive, nutritious, bland in flavour (non-sweet), highly soluble in cold water, and provides good flavour protection against oxidation. Maltodextrin has been proven to improve the oxidative stability of encapsulated oils, and is the most proper alternative for Arabic gum [29]. Maltodextrin provides a strong barrier against oxidation of core materials and protects against external factors [64]. The properties of the maltodextrin phase are also crucial in determining the rheological behaviour of the final product [65]. The carrier has advantages and disadvantages in terms of properties, costs and encapsulation efficiency. Different maltodextrins are classified into grades based on their dextrose equivalent (DE) value, which signifies the degree of hydrolysis of the starch molecule, and is directly linked to reducing sugar production. Maltodextrin with dextrose equivalent values of 10, 20, and 30 has desirable physical properties, and the particles have a smooth spherical surface [66,67]. Maltodextrins have been shown to be excellent thermal defenders, vital for protecting the integrity of anthocyanins during their encapsulation. A combination of proteins with different carbohydrates as wall materials, a blend of maltodextrin and sodium caseinate achieved a high encapsulation efficiency of some oils [32].

#### 5.3.2. Gums

Gums are usually applied during the encapsulation process for film forming due to their ability to stabilize the emulsions. One of the most frequently used gums is acacia gum, known as gum Arabic (GA), which has many desirable properties, such as its emulsification properties. GA is a polymer consisting of d-glucuronic acid, L-rhamnose, d-galactose, and L-arabinose, with almost 2% protein [68,69]. GA has functional properties including as an emulsifier, flavouring agent, humectant, thickener, surface-finishing agent and for retarding sugar crystallization [69]. GA is the most used gum in encapsulation technology, but suffers from restricted availability, high cost, and inability to prevent oxidation [60]. Several studies have reported the application of gum Arabic for the encapsulation of oils such as fish oil [70], kenaf seed oil [71], and palm fibre oil [35].

#### 5.3.3. Proteins

Proteins are excellent wall materials for encapsulation using spray drying due to their functional properties. Moreover, proteins provide high binding ability for flavour and oil compounds [29]. Proteins, in particular whey protein and sodium caseinate, have been studied as oil encapsulates due to their amphiphilic characteristics, which are caused by a hydrophilic group and a hydrophobic (or lipophilic) group, as well as their high diffusivity, which leads to better distribution around the enclosed oil surface [72,73]. There are several proteins or protein-containing isolates that have been applied for oil encapsulation, including soy, whey, casein and lecithin. A prevalent combination of oil encapsulation by spray drying is blend-based proteins, gums, and carbohydrates, in which the protein portion behaves as an emulsifier and carbohydrates act as the matrix-forming material. Specifically, soy or whey protein with maltodextrins are frequently applied, generally due to the variety of maltodextrins DE [74]. Among dairy proteins, sodium caseinate is preferred over other proteins due to its high solubility in water, emulsifying properties with oil and rapid formation of interfacial films and excellent surface activity [75]. Sodium caseinate has been reported to be the most effective emulsion stabilizer for fats. Sodium caseinate has been used alone or in blends with other wall materials to encapsulate a variety of volatile and non-volatile oils [76]. Research on the use of sodium caseinate as a wall material reports that it demonstrates good encapsulating properties, especially when used in combination with carbohydrates [77]. Sodium caseinate mixed with sunflower oil resulted in high encapsulation efficiency which was 99% in powder form [78]. According to Rosida et al. [79], using sodium caseinate to produce a non-dairy creamer resulted in a stable emulsion with white colour and significantly affected the protein content, giving a taste of milk (milk-sense). The mixture was concentrated and provided a white colour and stable emulsion when added to drinks or coffee.

Lecithin has been successfully used for the production of several encapsulated oils as an emulsifying agent to ensure the stability of the feeding emulsions before spray drying, for example, linseed oil [80] and kenaf seed oil [81]. Due to its lack of emulsifying properties, oil retention, and emulsion stability resulting from using maltodextrin alone as wall material, the addition of soy lecithin and carboxymethyl cellulose (CMC) improves encapsulation efficiency and oxidative stability [82].

## 6. Applications of Encapsulated Oils in Food

Consumer demand for healthy foods is growing rapidly due to scientific evidence that they improve human health. Furthermore, due to public concern about health, the demand for a healthy diet is increasing with the aim of reducing mortality and improving life quality. In general, the major difficulty in producing healthy oils for food applications is associated with the susceptibility of oils to oxidation. Several factors cause rapid oxidative degradation of food products that contain oils, including light, heat, and oxygen. Food deterioration caused by fatty acids limits the applications of some oils in food products, for example fish oil, which affects the texture, flavour, aroma, colour, and shelf-life of the product [83]. These drawbacks can be resolved via the encapsulation process of the oils to produce a stable powder with highly favourable properties. A wide range of seed and marine oils have been applied in food manufacturing to obtain functional food products with stable properties in the final product (Table 3). Examples of foods recently produced using encapsulated oils include dairy and non-dairy products, meat products, pastries, soups, etc. Several previous studies have reported the same limitation when applying fish oil and vegetable oils in food production, in that they are highly susceptible to oxidation. In addition, fortifying foods with some oils, particularly marine oils, is challenging due to their unpleasant taste, which could be overcome by applying the microencapsulation technique and masking the fishy taste. Moreover, replacing fats in some products with healthy sources of encapsulated oils is an area of interest for both manufacturers and consumers. Researchers have successfully incorporated encapsulated oils in various food products and observed higher oxidative stability, and high sensory acceptability for the products (Table 3).

Converting the liquids into a powdered encapsulated form can improved the stability and flow properties of the product during processing and handling. This technique may involve numerous types of functional oils for use as functional food ingredients [15].

## 7. Conclusions

Vegetable oils contain health promoting lipids with biological and functional properties. These oils are prone to oxidation, instability, and degradation due to the high content of unsaturated fatty acids. Converting the oils into a powder enhances their oxidative stability and allows alternative use of the ingredients. Selection of an appropriate encapsulation technique depends on the characteristics of the oil (core) compounds, the level of stability of the encapsulated oil under storage and processing conditions, the characteristics of the food components, the production cost, and the maximum encapsulation efficiency in the powder. The spray drying technique is an essential processing technology that is applied in many functional food, nutraceutical and pharmaceutical products. However, optimizing the conditions is highly recommended for the production of stable and high-quality encapsulated oils. In addition, there has recently been remarkable demand for functional foods enriched with bioactive compounds, including encapsulated functional oils from plants and marine life, which have successfully been launched into the market. Therefore, further investigations should be carried out to develop innovative materials and techniques in order to produce novel products fortified with encapsulated functional oil ingredients in the near future.

## Figures and Tables

**Table 1:** Optimization of spray drying process conditions for various types of oils.

Core Material	Wall Material	Total Solids	Inlet/Outlet Temperature	Reference
Palm fibre oil	Gum Arabic	20–40%	130–202 °C/NM	[35]
Sour cherry oil	Maltodextrin + gum Arabic	16.59–33.41%	120–220 °C/NM	[36]
Walnut oil	SMP + Tween 80	30%	180 °C/NM	[39]
Almond oil	Isolated starch	30–40%	145 °C/NM	[40]
Fish oil	Whey protein	30%	160 °C/NM	[41]
Corn oil	Brea gum	30%–40%	150 °C/60 °C	[42]
Virgin coconut oil	Soy protein isolate + maltodextrin	20 to 30%	160 and 180 °C	[43]
Palm Fibre Oil	Gum Arabic	35%	166 °C	[35]
PUFA-rich vegetable oil	Maltodextrin + modified starch	2:1 (wall: oil)	150 and 180 °C	[44]
Fish oil	Soybean protein	1:1, 2:1, 3:1, 4:1	180 °C/96 °C	[2]
Rapeseed oil	Soy protein isolate + Maltodextrin	30%	140–220 °C/NM	[45]
Squash seed oil	Maltodextrin + gum Arabic	25%,30%,35%	140, 160, 180 °C/ 90 °C	[46]
Echium oil	Gum Arabic	30%	150 °C/NM	[47]
*Nigella sativa* oil	Maltodextrin + Sodium Caseinate	20–60%	150–190 °C/85 °C	[48]
*Nigella sativa* oil	Maltodextrin + gum Arabic	30%	160 °C/88 °C	[31]
*Nigella sativa* oil	Maltodextrin + sodium octenyl succinic starch	25%	140 °C/95 °C	[49]
Lavender oil	Maltodextrin + gum Arabic	25%,30%,35%	140 °C/95 °C	[50]
Pomegranate Seed Oil	Xanthan gum + gum Arabic	30%,35%45%	170 °C/85 °C	[51]
Gac peel oil	Whey protein + gum Arabic	24.5%	160 °C/NM	[52]
Fish oil	Chitosan + maltodextrin	26.5%	160, 170, 180 °C/NM	[53]
Fish oil	Soy protein isolate + maltodextrin	45%	160 °C/85 °C	[54]
Rice bran oil	Jackfruit seed starch + whey protein isolate	30%	140, 150 and 160 °C	[55]
Citronella oil	Gum Arabic	20–60%	136–203 °C	[56]
Ginger oil	Inulin + whey protein isolate	20%,25%,30%	140 °C,155 °C and 170 °C	[57]

**Table 2:** Wall materials commonly used in the spray drying process.

Wall Material	Interest
Maltodextrin (DE < 20)	Film forming
Corn syrup solid (DE > 20)	Film forming, reducability
Modified starch	Very good emulsifier
Gum Arabic	Emulsifier, film forming
Modified cellulose	Film forming
Gelatin	Emulsifier, film forming
Cyclodextrin	Encapsulant, emulsifier
Lecithin	Emulsifier
Whey protein	Good emulsifier
Hydrogenated fat	Barrier to oxygen and water
Chitosan	Carrier of drug delivery

**Table 3:** Applications of encapsulated oils using the spray drying technique in several functional food products.

Encapsulated Oil	Product	Oil Source	Results	Reference
Fish oil	Burger	Marine	Burgers with microencapsulated fish oil showed the best scores for sensory traits and were stable during storage, and the thermal behaviour of the microparticles was similar before and after incorporation into the cookies.	[84]
Palm oil	Milk powder	Vegetable	The powders were easily soluble in water with low and non-hygroscopic moisture and low cohesiveness, which correspond to good flowability.	[85]
Chia oil	Cookies	Seed	Partial substitution of margarine by microencapsulated chia seed oil at 15 wt.% showed the best scores for sensory evaluation.	[86]
Flaxseed oil	Breads	Seed	Breads fortified with microencapsulated flaxseed oil showed lower peroxide index and higher α-linolenic acid value and helps preserve sensory properties compared to breads fortified with free flaxseed oil.	[87]
Canola oil	Non-dairy powder	Seed	Sodium caseinate and lactose via the Maillard reaction improved the encapsulation efficiency of oil up to 95.2%.	[88]
Shrimp oil	Biscuits	Marine	Biscuits fortified with 6% microencapsulated shrimp oil were stored in the dark to ensure their oxidative stability.	[89]
Fish oil	Chicken nuggets	Marine	Chicken nuggets enriched with microencapsulated fish oil showed no difference from control samples with respect to sensory attributes, and lower levels of lipid and protein oxidation were found microencapsulated fish oil.	[90]
Astaxanthin oil	Powder-based product	Marine	The encapsulation efficiency of astaxanthin powder was higher than 90%, the bioaccessebility of the reconstitution was around 80%, and it was stable under storage conditions.	[91]
Rapeseed oil	Yoghurt	Seed	Yoghurt matrix with microcapsules presented high acceptability of appearance and showed stability for 30 days.	[92]
Flaxseed oil	Chicken sausages	Seed	Spray-dried flaxseed oil formulations had lower values for cook loss and behaved differently during heating than the other formulations.	[93]
*Nigella sativa* oil	Non-dairy creamer	Seed	Microencapsulated oil demonstrated desired properties with high sensory acceptability for the revealed that developed non-dairy creamer.	[94]
Fish oil	Sausages	Marine	Fish oil microcapsules in cooked and dry-cured meat products labelled as “source of omega-3 fatty acids”, overall quality of the meat products enriched seems not to be impaired after storing.	[95]
*Nigella sativa* oil	Yoghurt	Seed	High stability of thymoquinone and proper chemical and sensory properties for yoghurt with *Nigella sativa* seeds oil microcapsules.	[49]
Fish oil	Sausage	Marine	The lipid oxidation increased, lipid reformulation increased MUFAs and n-3 PUFAs levels with highest TBARS values.	[96]
Tigernut, chia and linseed oils	Pâtés	Vegetable oils	Pâtés with microencapsulated oils showed modified fatty acid composition, decreasing the total amount of SFA and increasing PUFA (chia and linseed pâtés) or MUFA contents (tigernut pâtés).	[97]
Chia oil	Burgers	Seed	Microparticles of chia oil increased the terpenic volatiles and were characterized by the descriptors herbal and pleasant aroma and ideal texture with liking scores for sensory evaluation.	[98]
Fish oil	Chicken sausages	Marine	The sausages with the addition of microcapsules was characterized by higher values on the smell and consistency parameters with better results in the sensory evaluation.	[99]
Fish oil	Soup powder	Marine	The fortified soup powder of microencapsulated fish oil scored high in terms of sensory acceptance, proving its acceptability.	[100]
Catfish oil	Mushroom cream soup	Marine	Best physical characteristics of instant mushroom cream soup were reached with the addition of microcapsules at 3.6%.	[101]
